# The Advancement of Medicine by Research

**Published:** 1919-01-18

**Authors:** 


### The Hospital

The Workers' Newspaper of Administrative Medicine and Institutional Life, Administration, National Insurance and Health.

No 1702, Vol. LXV. SATURDAY, JANUARY 18, 1919. PRICE TWOPENCE

### THE ADVANCEMENT OF MEDICINE BY RESEARCH.

"Medicine" wrote Lord Bacon, "is a science which hath been more professed than laboured, and yet more laboured than advanced." This comment, to judge from some of the modern censors of the profession, could fairly be repeated at the present day, and out of it have emerged in various quarters, suggestions, or indeed demands, for the introduction of new methods in medical work. The development most generally proposed finds expression in such a phrase as the "organisation of research," and some of the critics do not hesitate to assure the public that were this formula only put into practical application, many of the most prevalent of the ills which afflict mankind would promptly be banished from our midst. To judge from the tone in which the advice is phrased "research" is quite a novel development, and its fruits, without question, will be early, certain, and abundant. The process is to be widely spread, and even the medical student is to take an active part in the solution of what are named "the elementary problems," though what exact meaning is to be attached to this phrase rests without definition. Some of the reformers find in the war the influence which has led to the discovery of the new method, while others look for its full expansion to the Ministry of Health. But all are agreed that it will cost a great deal of money, and some are quite ready to indicate the channels along which the financial stimulus ought to be directed. There is, in short, a more or less widespread conviction that a more successful struggle ought to be made against certain of the diseases from which man suffers, and that this struggle would become possible were the causes and conditions of such diseases more fully understood.

It may be agreed without qualification that for the position just presented there is a measure of justification, and if the argument is wisely directed much practical good will follow. The tendency of enthusiasm is to overstate its case and such an influence is easily recognised in the present instance. Some of the critics show a lamentable ignorance of medical work and history, and have yet to learn that "research" is not quite so new a method as they appear to imagine. Equally, if they were better acquainted with the past they would perhaps be less confident in their picture of the future. But neither of these considerations need exclude a wise policy from utilising their zeal and their optimism, for a driving power is needed as well as a method. Especially will such a power be required if, as is at least possible, the early and certain results now so lavishly promised to the public fail to realise themselves. The search for knowledge has almost always been a slow process, and practical application has not usually been the direct and inspiring motive. History, here, is likely to repeat itself, and if "research" is to be called on to justify itself and its expenditure by early and serviceable contributions it may fail to respond to the test. The true claim is not that great advances are inevitable if only the new panacea is adopted, but rather that the pursuit of knowledge is the appropriate expression of an intellectual curiosity and that in instances not a few the quest has discovered truths of considerable service in "the relief of man's estate."

What the war has done in the matter here discussed is to multiply in a certain number of diseased conditions, the opportunities for observation open to one and the same observer or group of observers, and, further, through the agency of military discipline, to organise these opportunities to the best advantage. By gathering together into a single institution a great group of patients suffering apparently from kindred disturbances it has enabled the physicians concerned to form a complete clinical picture of the malady, while the machinery at the disposal of the authorities has rendered possible careful enquiries and investigations relative to causation as well as the recording of outcomes and results.

The mass of information on particular points has been increased, and more complete and more searching records have been possible. In all this there is nothing new in principle, for observation and record have been the methods of clinical medicine from the earliest times. But an intensive element has been introduced, and the question is whether a similar force can be secured for the investigation of diseases as these occur in the ordinary conditions of civil society. Something of this order does, indeed, already exist in certain special hospitals, with, however, this important difference, that the medical staff are not, as are the military doctors, paid servants of the State, but are voluntary unpaid officials. Hence their services to the hospitals are only one part of their daily work, and they must needs live from some other source. That great advances in professional knowledge and capacity have, by the genius and labours of individuals, been accomplished in spite of these difficulties is beyond question, and it may fairly be admitted that a better organisation both of opportunities and workers may increase the number of such achievements. But to believe that the multiplication of machinery is certain to lead to a correspondingly increased output of advances and discoveries is a creed which has little basis in fact and experience. Not all endowment of research has been fruitful in result. What experience teaches is that to secure some commanding truth there is needed not only care and patience and diligence in the collection of facts, but also a certain capacity for vision and mental illumination. Such a gift cannot be created by subsidies or endowments, nor can it be wholly suppressed by their absence. There is every reason to say that "research" should be encouraged, but not every one who boasts the formula will enter into the kingdom.

